# Virtual Prospection of Marine Cyclopeptides as Therapeutics by Means of Conceptual DFT and Computational ADMET

**DOI:** 10.3390/ph15050509

**Published:** 2022-04-22

**Authors:** Norma Flores-Holguín, Juan Frau, Daniel Glossman-Mitnik

**Affiliations:** 1 Laboratorio Virtual NANOCOSMOS, Departamento de Medio Ambiente y Energía, Centro de Investigación en Materiales Avanzados, Chihuahua 31136, Mexico; norma.flores@cimav.edu.mx; 2 Departament de Química, Facultat de Ciènces, Universitat de les Illes Balears, E-07122 Palma de Malllorca, Spain; juan.frau@uib.es

**Keywords:** apratoxins, chemical reactivity theory, conceptual DFT, global and local reactivity descriptors, pKa, bioavailability, bioactivity scores, ADMET

## Abstract

Bioactive peptides are chemical compounds created through the covalent bonding of amino acids, known as amide or peptide bonds. Due to their unusual chemistry and various biological effects, marine bioactive peptides have garnered considerable research. The effectiveness of a bioactive marine peptide is attributed to its structural features, such as amino acid content and sequence, which vary depending on the degree of action. Cyclic peptides combine several favorable properties such as good binding affinity, target selectivity and low toxicity that render them an attractive modality for the development of therapeutics. The apratoxins are a class of molecules formed by a series of cyclic depsipeptides with potent cytotoxic activities. The objective of this research is to pursue a computational prospection of the molecular structures and properties of several cylopeptides of marine origin with potential therapeutic applications. The methodology will be based on the determination of the chemical reactivity descriptors of the studied molecules through the consideration of the Conceptual DFT model and validation of a particular model chemistry, MN12SX/Def2TZVP/H_2_O. These studies will be complemented by a determination of the pharmacokinetics and ADMET parameters by resorting to certain cheminformatics tools.

## 1. Introduction

The chemical compounds known as bioactive peptides are created by amino acids joining together through covalent connections, which are known as amide or peptide bonds. Although some bioactive peptides can be found in their natural state, the vast majority of known bioactive peptides are encoded in the structure of the parent proteins and are only released through enzymatic activities, which account for the majority of their release. Due to their unusual chemistry and various biological effects, marine bioactive peptides have garnered considerable research. A bioactive marine peptide’s effectiveness is attributed to its structural features, such as amino acid content and sequence, which vary depending on the degree of action [1,2,3].

Computational Medicinal Chemistry (CMC) has established itself as a critical component of contemporary drug discovery. The goal of CMC is not to replace in vitro or in vivo tests, but rather to accelerate the discovery process, reduce the number of candidates that must be evaluated experimentally, and rationalize the selection of those candidates that are investigated [4,5]. Density Functional Theory (DFT) represents a computational methodology that has gained popularity in recent years for calculating molecular properties. DFT has been extensively used to compute the electronic structure and properties of molecules in both the ground and excited electronic states and in both the gas and aqueous phases. Conceptual DFT (CDFT), as a specialized branch of DFT, was created to develop a chemical reactivity theory on the basis of certain chemical concepts that arise from DFT by expressing their properties in terms of reactivity descriptors. It has been successfully used for the study of the chemical reactivity of atoms, molecules, and organic and metallic clusters [6,7,8,9,10,11,12,13,14,15,16,17].

There are several other critical parameters in the development of new drug molecules that contribute to the definition of overall safety margins, dose intervals, and dose amounts. These parameters include absorption, distribution, metabolism, excretion, and toxicology (ADMET). The comprehensive investigation of physicochemical parameters is crucial as medicinal chemists can easily draw links to such physicochemical parameters. When developing a new medicine, the optimal balance of physicochemical qualities and ADMET criteria must be achieved. The research related to these properties using Computational Chemistry and Molecular Modeling techniques has been labelled as Computational ADMET and is being increasingly considered within the context of drug discovery and design (http://crdd.osdd.net/admet.php (accessed on 22 February 2022)).

**Figure 1:**
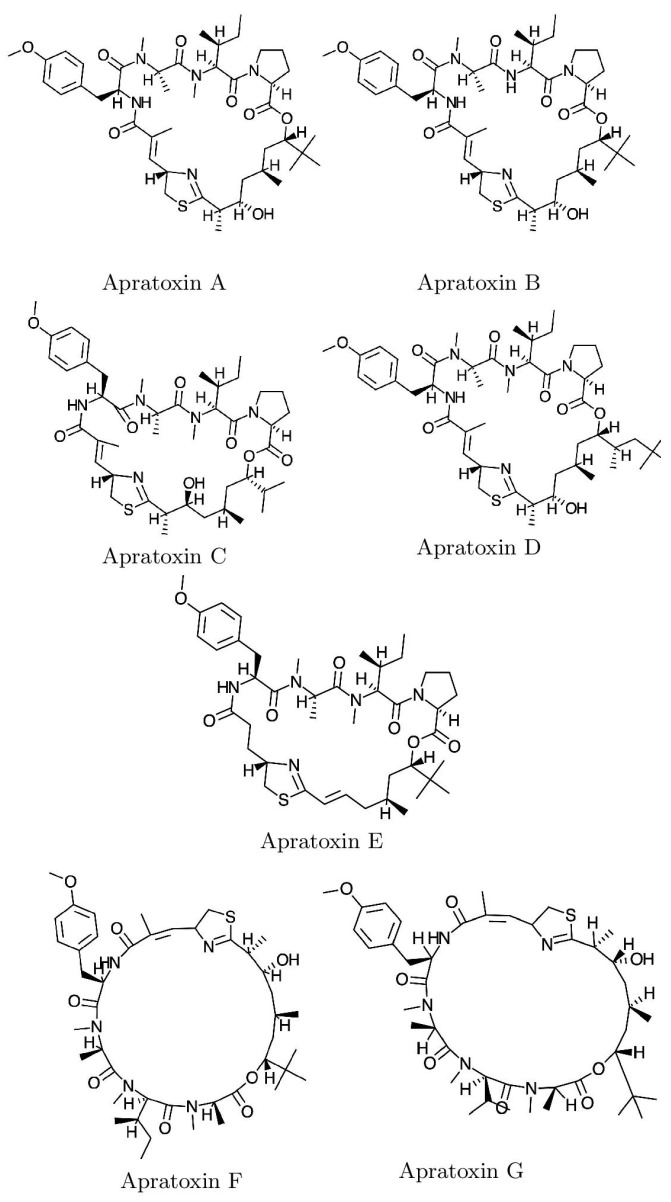
Graphical sketches of the molecular structure of Apratoxin A, Apratoxin B, Apratoxin C, Apratoxin D, Apratoxin E, Apratoxin F and Apratoxin G.

The objective of this research is to pursue a computational prospection of the molecular structure and properties of several cylopeptides of marine origin with potential therapeutic applications. Cyclic peptides combine several favorable properties such as good binding affinity, target selectivity and low toxicity that make them an attractive modality for the development of therapeutics. The apratoxins represent a class of molecules formed by a series of cyclic depsipeptides with potent cytotoxic activities, usually in the nanomolar range, and characterized by a thiazoline unit and an extensive polyketide-derived moiety as part of the macrocyclic5 structure [18]. Apratoxins A–C are cyclodepsipeptides isolated from *Lyngbya majuscula* with in vitro cytotoxicity against LoVo and KB cell-lines [19]. Apratoxin D is a potent cytotoxic cyclodepsipeptide from Papua New Guinea collections of the marine cyanobacteria *Lyngbya majuscula* and *Lyngbya sordida* [19]. The peptide apratoxin E was also isolated from *L. bouillonii*, and was found to bear superior cytotoxicity when compared to its closest analog, the semi-synthetic E-dehydroapratoxin A, against various cancer cell-lines derived from bone, colon, and cervix, with ranging values of activity but less active when compared to Apratoxin A [20]. Apratoxins F and G are two cytotoxic cyclic depsipeptides which were isolated from *Lyngbya bouillonii* collected from Palmyra, characterized by the presence of an N-methyl alanine residue at a position where earlier Apratoxins contained a proline unit [21,22]. Graphical sketches of the molecular structures of the Apratoxins A–G as retrieved from the PubChem database (https://pubchem.ncbi.nlm.nih.gov (accessed on 22 February 2022)) are shown in Figure 1. The methodology will be based on the determination of the chemical reactivity descriptors of the studied molecules through the consideration of the Conceptual DFT model and validation of a particular model chemistry, MN12SX/Def2TZVP/H_2_O, through an earlier proposed procedure [23,24,25,26,27]. These studies will be complemented by a determination of the pharmacokinetics and ADMET parameters by resorting to certain chemoinformatics tools.

## 2. Results and Discussion

**Figure 2:**
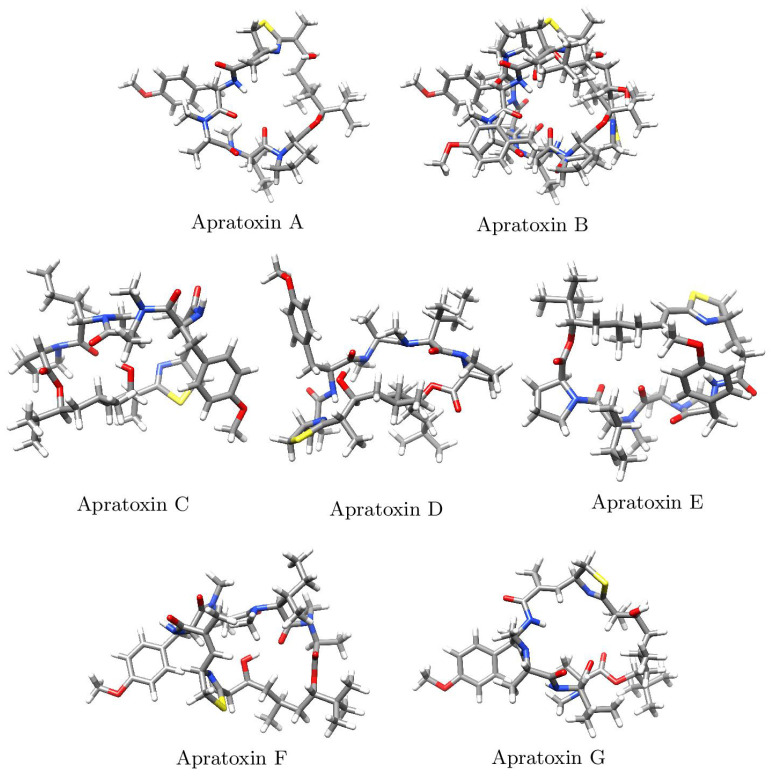
Optimized molecular structures of Apratoxin A, Apratoxin B, Apratoxin C, Apratoxin D, Apratoxin E, Apratoxin F and Apratoxin G.

The Kohn–Sham (KS) methodology includes the determination of the molecular energy, electronic density and orbital energies of a given system, in particular, the frontier orbitals HOMO and LUMO which are intrinsically related to the chemical reactivity of molecules [28,29,30,31]. Through the comparison of a density functional’s results to experimental values or high-level computations, one can assess the quality of the density functional. A methodology referred to as KID was developed by our research group [23,24,25,26] to avoid these comparisons and to validate the ability of a given density functional in the fulfilment of the Janak and the Ionization Energy Theorems [32,33,34,35,36]. This connection between $\epsilon_{H}$ to −I and $\epsilon_{L}$ to −A, is verified through the formulas J${}_{I}$ = $\epsilon_{H}$ + E${}_{gs}$(N − 1) − E${}_{gs}$(N), J${}_{A}$ = $\epsilon_{L}$ + E${}_{gs}$(N) − E${}_{gs}$(N + 1), and J${}_{HL}$ = $\sqrt{{J_{I}}^{2}+{J_{A}}^{2}}$, with $\epsilon_{H}$ and $\epsilon_{L}$ representing the HOMO and LUMO energies related to the marine cyclopentapeptides considered in this research. An extra KID descriptor ΔSL, equal to the difference in energies between the SOMO (corresponding to the radical anion’s HOMO) and the neutral system’s LUMO, was devised to aid in the verification of this methodology’s accuracy [23,24,25,26]. The results for these calculations are presented in Table 1 while the corresponding optimized molecular structures are displayed in Figure 2.

As can be appreciated by the inspection of Table 1, the use of the MN12SX/Def2TZVP/H_2_O model chemistry is justified since the KID procedure shows the fulfillment of the Janak and Ionization Energy theorems for all molecular systems considered in this research.

This methodology is convenient when considering quantitative qualities related to Conceptual DFT descriptors [6,7,8,9,37,38]. The definitions for the global reactivity descriptors are [6,7,8,9,37,38]: Electronegativity as $\chi \approx \frac{1}{2}\left(\epsilon_{L}+\epsilon_{H}\right)$, Global Hardness as $\eta \approx (\epsilon_{L}-\epsilon_{H})$, Electrophilicity as $\omega \approx {\left(\epsilon_{L}+\epsilon_{H}\right)}^{2}/4(\epsilon_{L}-\epsilon_{H})$, Electrodonating Power as $\omega^{-}\approx {\left(3\epsilon_{H}+\epsilon_{L}\right)}^{2}/16\eta$, Electroaccepting Power as $\omega^{+}\approx {\left(\epsilon_{H}+3\epsilon_{L}\right)}^{2}/16\eta$ and Net Electrophilicity as $\Delta \omega^{\pm }=\omega^{+}-\left(-\omega^{-}\right)=\omega^{+}+\omega^{-}$. These global reactivity descriptors that arise from Conceptual DFT [6,7,8,9,37,38], have been complemented by the Nucleophilicity Index N [39,40,41,42,43]. The results for the determination of the Conceptual DFT reactivity descriptors for the selected peptides are displayed in Table 2.

Analysis of the Conceptual DFT descriptors reveals some information about the stability, electrophilicity, and nucleophilicity of the compounds under investigation. It can be appreciated form Table 1 and Table 2, that Apratoxin A exhibits the largest global hardness (or HOMO-LUMO gap) among the molecules. Thus, it will be the least reactive of the studied peptides, and in turn, Apratoxin C will be the most reactive. Apratoxins B and G display the highest electronic chemical potentials and can efficiently exchange electron density with the environment. By studying the electrophilicity of a series of reagents involved in Diels–Alder reactions [41,44,45], an electrophilicity ω scale for the classification of organic molecules as strong, moderate or marginal electrophiles was proposed with ω> 1.5 eV for the first case, 0.8 <ω< 1.5 eV for the second case and ω<0.8 eV for the last case [41,44,45]. By inspection of Table 2, it can be observed that with the exception of Aparatoxin A, the other peptides may be regarded as moderate electrophiles. Organic molecules can be classified as strong (N > 3 eV), moderate (2.0 eV ≤ N ≤ 3.0 eV), and marginal nucleophiles (N < 2.0 eV) in polar organic reactions [40]. From Table 2, it can be concluded that all peptides under investigation may be considered moderate nucleophiles.

A simple QSAR relationship pKa = 16.3088 − 0.8268 η was employed to determine the pKa of cyclopeptides using the methods provided earlier, which has proved useful in the research of amino acids and short peptides, as well as in the creation of Advanced Glycation End Products (AGEs) inhibitors [46]. These results, together with some pharmacokinetic parameters of utility in the process of drug design and discovery, are reported in Table 3.

Although this research deals with the use and validation of certain computational techniques applied in the determination of the chemical reactivity properties of the studied molecules, it would be desirable to identify some correlation between the Conceptual DFT descriptors and the pharmacokinetics and ADMET indices, as demonstrated for the case regarding pKas. However, there is no sense in identifying QSAR relationships when working with only seven molecules. Some qualitative correlations can be mentioned instead. For example, it can be seen from Table 3, that Apratoxin D exhibits the largest value of logP, but is also among the peptides with the lowest values of electrophilicity ω (Table 2). This opens the way for identifying QSAR relationships in future studies with a larger number of molecular systems.

**Figure 3:**
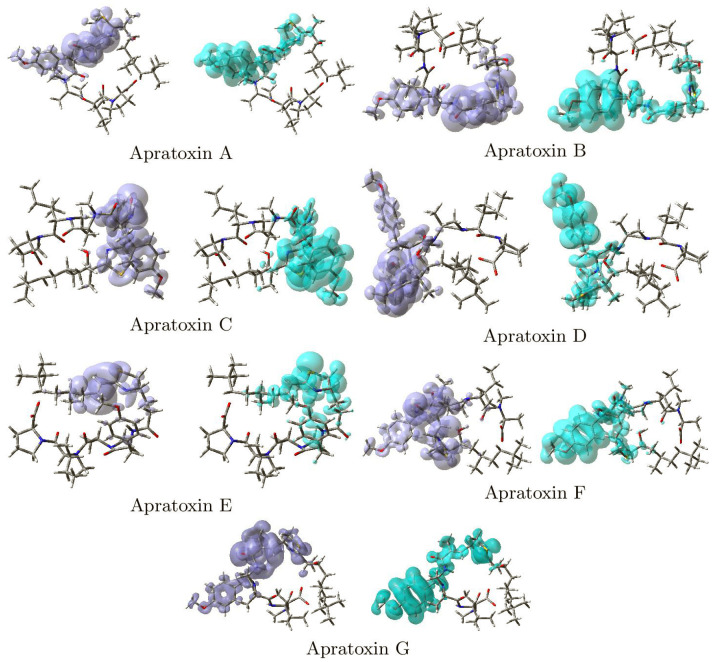
Graphical representation of the Dual Descriptor DD of the Apratoxin A, Apratoxin B, Apratoxin C, Apratoxin D, Apratoxin E, Apratoxin F and Apratoxin G molecules. Left: DD > 0, Right: DD < 0.

The Dual Descriptor DD is a local reactivity descriptor defined as DD = (∂f(r)/${\partial \;N)}_{\upsilon (r)}$ [10,47,48,49,50,51]. A molecule’s nucleophilic and electrophilic sites can be defined using the Dual Descriptor DD without any confusion [51]. To further comprehend the local chemical reactivity of Apratoxins A–G, the Dual Descriptor DD for these compounds is shown graphically in Figure 3.

The estimated Bioactivity Scores of the Apratoxins A–G family of marine cyclopeptides are displayed in Table 4. The interpretation of the presented results indicates that all members of the Apratoxin family (with the exception of F and G) will behave as protease inhibitors. Furthermore, Apratoxins A, B, C and E could act as GPCR ligands.

The computed ADMET pharmacokinetic profiles of the Apratoxins A–G family of marine cyclopeptides are presented in Table 5.

As the information presented is given in terms of positive and negative descriptors, and through numerical values, it is not possible to identify QSAR relationships between the ADMET properties and the Conceptual DFT reactivity descriptors. From Table 5, it can be appreciated that all the members of the Apratoxin family of cyclopeptides will display good gastrointestinal absorption but not BBB permeability. All the molecules could behave as P-gp substrates and also act as P-gp I inhibitors and with the exception of Apratoxin E, as P-gp II inhibitors. Their behavior relating to the different variants of cytochrome p450 will differ as displayed in Table 4 with some particularities for each of the variants. AMES toxicity will not occur for any of the studied peptides, although hepatoxicity will be displayed. All peptides will exhibit negative behavior as inhibitors of hERG I and II, with the exception of Apratoxin F regarding hERG II inhibition. Finally, none of the cyclopeptides will generate skin sensitization.

## 3. Materials and Methods

### 3.1. Conceptual DFT Studies

The main approach of our research is based on the application of Conceptual DFT [6,7,8,9,10,11,12] for the prediction of the chemical reactivity properties of the studied molecules. The starting point is the calculation of their fundamental molecular structures determining the electronic densities and from these the corresponding molecular and orbital energies, mainly the Highest Occupied Molecular Orbital (HOMO) and the Lowest Unoccupied Molecular Orbital (LUMO). As usual, the many conformers of the studied peptides will be predicted considering the MarvinView 17.15 software from ChemAxon (http://www.chemaxon.com (accessed on 22 February 2022)). This will be achieved with the help of the MMFF94 force field for performing Molecular Mechanics calculations. Every selected conformer for each peptide will be subject to a geometry optimization and frequency calculation by means of the Density Functional Tight Binding (DFTBA) methodology [52] for the obtention of suitable starting molecular structures. This will be followed by geometry reoptimization, frequency analysis and calculation of the electronic properties and the chemical reactivity descriptors of the cyclopeptides considering the MN12SX/Def2TZVP/H_2_O model chemistry [53,54,55] within the context of the Kohn–Sham (KS) approach [28,29,30,31]. The absence of imaginary frequencies will be checked as a guarantee that the optimized structures may be considered as minima within the energy landscape. Gaussian 16 software [52] and the SMD solvation model [56] will be considerd owing to the fact that the chemistry of the chosen model was previously proved to fulfil the ’Koopmans in DFT’ (KID) procedure [23,24,25,26]. This methodology is useful to verify whether a given density functional behaves according to the Janak and Ionization Energy theorems. It has been previously shown [27] that while in the absence of a solvent, the calculations performed with the ωB97XD density functional fulfil these theorems, when in the presence of water as a solvent, the performance of the MN12SX density functional is much better.

### 3.2. Computational ADMET

It is crucial to understand pharmacokinetics, or the fate of a given molecule in the body, during the drug research and design process. This can be estimated in terms of individual indices collectively known as ADMET (absorption, distribution, metabolism, excretion, and toxicity). Computer models are frequently used as an alternative to experimental methods for establishing these parameters. Chemicalize, a software developed by ChemAxon (http://www.chemaxon.com (accessed on 22 February 2022)), was considered for this purpose, while more involved additional information regarding pharmacokinetic parameters and ADMET properties were obtained using admetSAR [57], a software for the prediction of these properties using SMILES (http://lmmd.ecust.edu.cn/admetsar2/ (accessed on 22 February 2022)). Molinspiration software (https://www.molinspiration.com/ (accessed on 22 February 2022)) was used to compute numerous molecular characteristics and forecast the bioactivity scores for pharmacological targets such as enzymes and nuclear receptors, kinase inhibitors, GPCR ligands, and ion channel modulators.

## 4. Conclusions

Through the combination of a methodology based on the estimation of Conceptual DFT reactivity descriptors, a procedure for validating the fulfilment of the Janak and Ionization Energy theorems, and certain informatics tools aiding in the estimation of pharmacokinetic parameters and ADMET indices, information regarding the potential therapeutic properties of a family of cyclopeptides of marine origin has been reported. The results could provide the basis and starting point for future studies on experimental and clinical research concerning these interesting molecules. These conclusions pave the way for considering these Conceptual DFT reactivity indices as descriptors of bioactivity in future studies employing a larger number of potential therapeutic drugs.

## Figures and Tables

**Table 1:** Frontier orbital energies, HOMO-LUMO gap and the KID descriptors for the Apratoxins A–G family of marine cyclopeptides (all in eV).

Apratoxin	HOMO	LUMO	SOMO	H-L Gap	J${}_{I}$	J${}_{A}$	J${}_{HL}$	ΔSL
A	−6.24	−1.22	−1.18	5.02	0.025	0.019	0.032	0.038
B	−6.20	−1.74	−1.70	4.47	0.023	0.017	0.028	0.037
C	−6.04	−1.76	−1.73	4.28	0.091	0.016	0.092	0.029
D	−6.22	−1.38	−1.38	4.84	0.013	0.005	0.014	0.005
E	−6.08	−1.69	−1.67	4.39	0.117	0.007	0.117	0.015
F	−6.19	−1.40	−1.37	4.79	0.015	0.019	0.024	0.037
G	−6.12	−1.79	−1.77	4.33	0.100	0.010	0.100	0.022

**Table 2:** Global reactivity descriptors for the Apratoxins A–G family of marine cyclopeptides (all in eV, with the exception of S, in eV${}^{-1}$).

Apratoxin	χ	η	ω	S	N	$\omega^{-}$	$\omega^{+}$	$\omega^{\pm }$
A	3.73	5.02	1.39	0.20	2.56	4.95	1.22	6.17
B	3.97	4.47	1.76	0.22	2.59	5.79	1.82	7.61
C	3.90	4.28	1.78	0.23	2.75	5.78	1.87	7.65
D	3.80	4.84	1.50	0.21	2.57	5.19	1.39	6.58
E	3.88	4.39	1.72	0.23	2.72	5.65	1.76	7.41
F	3.80	4.79	1.51	0.21	2.60	5.21	1.41	6.62
G	3.96	4.33	1.81	0.23	2.67	5.86	1.91	7.77

**Table 3:** Predicted pharmacokinetic parameters of the Apratoxins A–G family of marine cyclopeptides.

Apratoxin	ΔG of Solvation	pKa	logP	TPSA	Molecular Volume
	(kcal/mol)			(Å${}^{2}$)	(Å${}^{3}$)
A	−25.86	12.90	5.49	158.16	809.91
B	−31.99	12.97	5.26	166.94	792.97
C	−27.41	12.90	5.11	158.16	793.67
D	−23.32	12.90	7.41	158.16	860.10
E	−26.30	12.47	6.21	137.93	768.72
F	−25.54	12.73	6.07	158.16	803.47
G	−31.00	12.76	5.54	166.94	769.72

**Table 4:** Bioactivity Scores of the Apratoxins A–G family of marine cyclopeptides.

Apratoxin	GPCR	Ion Channel	Nuclear Receptor	Kinase	Protease	Enzyme
	Ligand	Modulator	Ligand	Inhibitor	Inhibitor	Inhibitor
A	−1.95	−3.12	−3.14	−3.02	−1.11	−2.42
B	−1.73	−3.00	−2.97	−2.86	−0.92	−2.19
C	−1.75	−2.96	−2.92	−2.90	−0.96	−2.24
D	−2.69	−3.51	−3.59	−3.53	−1.85	−2.98
E	−1.46	−2.74	−2.69	−2.48	−0.79	−1.89
F	−3.77	−3.88	−3.88	−3.89	−3.67	−3.81
G	−3.76	−3.87	−3.87	−3.88	−3.65	−3.80

**Table 5:** Computed ADMET pharmacokinetic profiles of the Apratoxins A–G family of marine cyclopeptides.

	Apratoxins						
Property	A	B	C	D	E	F	G
HI Absorption	+	+	+	+	+	+	+
BBB Permeability	−	−	−	−	−	−	−
Caco-2	+	+	+	+	+	+	+
P-gp Substrate	+	+	+	+	+	+	+
P-gp I Inhibitor	+	+	+	+	+	+	+
P-gp II Inhibitor	+	+	+	+	−	+	+
CYP2D6 Substrate	−	+	−	−	−	−	−
CYP3A4 Substrate	+	−	+	+	+	+	+
CYP1A2 Inhibitor	−	+	−	−	−	−	−
CYP2C19 Inhibitor	−	−	−	−	−	−	−
CYP2C9 Inhibitor	−	−	−	−	−	−	−
CYP2D6 Inhibitor	−	−	−	−	−	−	−
CYP3A4 Inhibitor	+	−	+	+	+	+	−
OCT2 Substrate	−	−	−	−	−	−	−
AMES Toxicity	−	−	−	−	−	−	−
hERG I Inhibitor	−	−	−	−	−	−	−
hERG II Inhibitor	−	−	−	−	−	+	−
Hepatoxicity	+	+	+	+	+	+	+
Skin Sensitization	−	−	−	−	−	−	−

## Data Availability

Data is contained within the article.
